# Adoptees’ views and experiences of direct-to-consumer (DTC) genomic testing: an exploratory interview study from the UK

**DOI:** 10.1007/s12687-022-00622-y

**Published:** 2022-11-29

**Authors:** Alison C. Kay, Nicola V. Taverner

**Affiliations:** 1 The MRC Weatherall Institute of Molecular Medicine, University of Oxford, Oxford, UK; 2 School of Medicine, Cardiff University, Cardiff, UK

**Keywords:** DNA genealogy, Adoptee, Foundling, Genomics, Direct-to-consumer (DTC)

## Abstract

Direct-to-consumer (DTC) genomic testing for ancestry and health may appeal to adoptees looking to fill gaps in their family information. There are only a handful of published studies on adoptees’ views and experiences of DTC testing and none of these is from the UK. The recent UK House of Commons Science and Technology Committee report (GB Parliament, House of Commons 2021) did not address the gains or challenges for adopted people specifically, although the Committee did consider that robust evidence of opportunities or risks for any user of a DTC testing kit is limited. In this study presented here, semi-structured interviews were conducted with ten UK adult adoptees recruited via social media. Reflexive thematic analysis (Braun and Clarke 2006, 2019) of the interview transcripts identified three main themes: *Decisional influencers* of longing, uncertainty and normalisation of DNA kit use; *Informational drivers* to gain clarity but avoid new worrisome information; and talk around *Negotiating Visibility* to birth family and commercial third parties. A further theme of *Meaning Making* related to adoptees’ views of testing outcomes as bringing feelings of resolution or discordance. This study identified many challenging deliberations for adoptees in evaluating whether to take a DTC test and what to do when their results were returned. Additionally, adoptees’ consideration of data privacy issues appears hampered by already having shared identifying information about themselves in their wider adoptee search. Further research is encouraged.

## Introduction

Adopted adults who are disenfranchised from their family history can access their genetic information through direct-to-consumer (DTC) testing. Indeed, commercial testing companies have targeted them directly with their marketing (23andMe 2019; Ancestry 2019), including offers of free DNA testing kits in return for adding their DNA to a company’s database (My Heritage 2019) and proclamations of the positive benefits for the adopted (EasyDNA 2022). The UK has seen considerable market growth in this type of genomic consumerism, also referred to as recreational testing, particularly for family history purposes (Kennett 2017; Padilla & Border 2012; Schwartz 2019; Weinberg 2018). One company, 23andMe, has sold over 250,000 genomic testing kits in the UK as of June 2020 (GB Parliament, House of Commons 2021). There has been very limited research on how adult adoptees view and experience these tests.

A structured literature search strategy was carried out using GoogleScholar and PubMed and the keywords: “adopted”, “adopted adults”, “genetic testing” and also “DTC” and “Direct-to-Consumer”. The latter terms are now widely used by official bodies and medical journals to denote a range of genetic testing products available to purchase commercially and use in the customer’s home. Additional hand-searches were also undertaken using the reference lists of identified publications. Three studies with clear methodologies were identified, all of which focussed on the motivations of US adoptees (Baptista et al. 2016; Lee et al. 2021; Strong et al. 2017). The literature search also identified some debate as to whether the lack of a family health history represents a health disparity for adoptees — defined as a systematic, unjust and unavoidable disadvantage — and whether genetic/genomic testing could be a useful and ethical substitute (Casas 2018; Fullerton 2016; Lord 2018; May and Grotevant 2018; May et al. 2015; May et al. 2016a; May et al. 2016b; May & Fullerton 2021). No research on the views and experiences of UK adoptees was identified.

Adoptees may be concerned that they are unknowingly carrying a genetic condition that they could pass on or have already unknowingly passed on to their offspring. Traditional carrier screening is not a practical solution for adoptees because it assesses reproductivity risks relating to a single condition already known to be carried within a family. Adoptees may seek genetic health information to help fill health information gaps (May et al. (2015), May et al. (2016a), May et al. (2016b)) or because they perceive it may be helpful for reproductive decision-making (May and Grotevant 2018; Spencer et al. 2018). However, without knowledge of the association of any identified genetic variants with disease outcomes in biological relatives, this uncontextualised genetic information could create more uncertainty for the adoptee or lead to misunderstanding or false reassurance (Fullerton 2016; Lord 2018; Quintans et al. 2014; Ramos & Weissman 2018; Seward 2017). In the UK context specifically, concerns have been raised by the Association of Genetic Nurses and Counsellors (AGNC) about the limitations of DTC health tests, especially the potential for customers’ poor understanding of the meaning of their results (AGNC 2022). Adoptees may not be fully informed of this complexity when forming their views on whether to proceed with DTC testing and whether to purchase an ancestry or health kit, or indeed the combined bundle.

Studies of adoptees’ motivations for using DTC testing are limited. The three studies identified in peer-reviewed journals (Baptista et al. 2016; Lee et al. 2021; Strong et al. 2017) do support the assertion that adoptees view genetic information as valuable for their life planning and addressing gaps in their family history. There is some consistency in their findings, in particular relating to the desire by adoptees to address their own genetic risk, to be able to pass on this information to their biological children, and to be able to do all this with affordability and emotional convenience — for example avoiding a reunion. However, these studies differ significantly in their design and each has limitations for investigating adoptee perceptions of this consumer genetic technology.

Baptista et al. (2016) used a large and comparative sample from 23andMe and Pathway Genomics consumers (adopted *n* = 80, non-adopted *n* = 1527), but the study was not designed at the outset to investigate the views of adopted adults and participants were recruited from the customer database of DTC companies and hence were a self-selecting group. In contrast, Strong et al.’s (2017) sample (*n* = 17) was drawn from adoption groups rather than DTC customers and was less self-selecting for test-takers. The qualitative study design was appropriate for exploring perspectives because it enables the researchers to work closely with the participants (Coolican 2014; Forrester 2010) but Strong et al.’s (2017) focus groups comprised only individuals who had not yet made a decision about testing and hence their participants’ responses were speculative. Lee et al.’s (2021) recent quantitative study (*n* = 117) was designed to include both those who did and did not take DTC tests. As most adoptees in their study did proceed with testing, the authors reported they were not able to gain the intended comparative view.

These three studies on US adoptees included questions around data privacy and it is interesting that there is discordance between these findings. Whereas Baptista et al.’s (2016) study found that adoptees were less likely to consider genetic privacy than non-adoptees (23% v 41%; *p* = 0.001), Lee et al. (2021) and Strong et al. (2017) both identified concerns from adoptees about privacy and the misuse of their data. Looking at wider research, there are low levels of public awareness about data storage and sharing practices and this lack of information could impact particular groups, such as adoptees, differently (Aitken et al. 2017; McCormack et al. 2016). Despite low awareness, commercial DTC databases raise issues around confidentiality, an individual’s control over their data, the non-medical use of their data, who can access that data, and abuses of data (Aitken et al. 2017; Middleton 2018; GeneWatch UK 2017; Sandor 2018; Sorani et al. 2015). Hence, adoptees’ views and experiences around data privacy warrants further investigation.

Given the inconsistencies in the US findings and the lack of research on UK adoptees, this study sought to answer the research question: What do UK adult adoptees’ accounts reveal about their views and experiences of accessing their family history and family health history via DTC genetic testing? Its aim was to explore the benefits and harms with no hypothesis and to consider the specific role of data privacy.

## Method

A qualitative approach was chosen as the best fit for studies on perspectives (Coolican 2014; Forrester 2010). There are many secrecies and silences around adoption in the UK (Keating 2008) and an approach to data collection that enabled participants to speak freely was important. For this reason, one-on-one interviews were selected rather than focus groups because a group setting could have inhibited participants (Howitt and Cramer 2011). Remote interviewing using the audio-function of video software (Blackboard Collaborate) made it practical for participants from across the UK to take part and there is some evidence that not being face-to-face can be beneficial for sensitive topics, enabling participants to speak more freely (Howitt and Cramer 2011). The possibility of triggering difficult emotions or genetic health concerns was addressed in the Participant Information Sheet and contacts provided for a recognised UK counselling service (Samaritans) and a named Registered Genetic Counsellor.

Opportunity sampling was used for this study as this is particularly suitable for exploratory studies where the target population is very dispersed (MacFarlane et al. 2014; Robson 2011). The inclusion criteria were adults aged 18 and above and legally adopted in the UK. Those who had been adopted by a biological relative, were not yet 18 and did not speak fluent English were excluded. All participants were required to be able to consent in their own right. Cardiff University School of Medicine Research Ethics Committee approval was granted and the approved recruitment material, incorporating a QR link to a website hosting the Participant Information Sheet and Consent Form, was then circulated via social media (Twitter, Facebook and LinkedIn) at intervals across November 2020. Hashtags were utilised and key accounts of people interested in DNA genealogy and adoption were tagged to further circulate the advertisement. The social media posts were targeted towards both those that had and had not taken a DTC test for ancestry or health. An alternative email address for requesting the forms was also provided. All personal and interview data collected from those recruited was stored and protected according to GDPR and Cardiff University data storage policies.

The interviews were conducted using an ethics committee–approved interview guide, posing questions in a responsive manner to maintain a conversational flow (Coolican 2014; Forrester 2010). Ten interviews of approximately 1-h each were recorded and analysed, in accordance with the 10–15 suggested as sufficient in qualitative genetic counselling research (MacFarlane et al. 2014). The interviews were manually transcribed by the first author which presented an opportunity to gain familiarity with the data and to connect with the participant’s emphasis, tone and meaningful pauses (Howitt and Cramer 2011). Using reflexive thematic analysis to work with the transcripts then enabled the underlying meaning of statements to be analysed and identification of complexities not necessarily obvious to the participant themselves. Braun and Clarke’s 6-staged approach to thematic analysis (2006, 2019) was applied — an explicit and replicable method of analysis: familiarisation with the data, generating initial codes, searching for themes, reviewing themes, defining and naming themes, and producing the report (Braun & Clarke 2006, 2019). Sections of coding and themes were reviewed by the second author and the thematic map was then produced.

The authors of this article reflected on insider positionality as the primary researcher conducting the interviews is an adoptee. Disclosure of this positionality confers the potential for the benefits of openness, rapport, and the gathering of deeper reflections from participants (Chavez 2008; Ross 2017). However, to maintain empathic distance during the interviewing stage (Berger 2015), it was decided that this status would be volunteered only if the interviewer was directly asked and any further conversation around this was then directed until after the interview. To minimise personal bias during the analysis stages, the primary researcher utilised standard reflective practice models (Johns 1994; Rolfe et al. 2001) to aid the progress of the research and shared coding and thematic map development with the second author. Additionally, studies have reported that undertaking yarn craft can access a flow mental state (Ferry Palmer-Cooper 2021; Riley et al. 2013) useful to qualitative research, which by its nature is often iterative and conducted over a period of time (Ausband 2006). The first author accompanied the project with a crochet colour-story blanket.

## Results

Recruitment took place during November 2020, using Twitter, Facebook and LinkedIn. Twitter, in particular, achieved wide dissemination of the advertisement: 5 Tweets were viewed in 11,423 Twitter feeds and 398 people interacted with the tweet, including 21 retweets to extend the recruitment reach. The engagement rate for each of the original tweets was consistently high and well above 1% — generally considered very good engagement on social media platforms (Table 1) (LSE 2021).

**Table 1:** Recruitment reach (estimated)

Platform	Posts	Impressions (views)	Engagements (interactions, e.g. sharing)	Engagement rate (%)
Twitter	5	11,423	398	1.6–5.4
Facebook	1	3920*	N/A	N/A
LinkedIn	1	66	N/A	N/A
Combined	7	15,409	398	1.6–5.4

*Based on combined “friends” of those who shared the Facebook post. This may contain duplicates and is therefore a rough estimate.

Twelve adoptees responded to the social media advertisements, 10 of whom returned a signed consent form. Interviews of approximately 1-h duration were conducted during November and December 2020. Seven of the 10 participants had taken a DTC test before their involvement in this study. The study participants consisted of 6 women and 4 men (Table 2).

**Table 2:** Sample characteristics

Pseudonym	Sex	Age	DNA tests	Extra info
Dolly	Female	61	23andMe, Ancestry	Foundling* (Hong Kong)
Sally	Female	46	None	
Jonathan	Male	39	Ancestry	
Pippa	Female	58	Ancestry, 23andMe	
Karen	Female	65	Ancestry	
Robert	Male	49	23andMe	
Carl	Male	57	None	Twin
Stephan	Male	54	Ancestry	British Adoption Project
Janet	Female	73	None	
Tanya	Female	61	23andMe	Foundling (Hong Kong)

*A foundling is a person who was abandoned by their birth parents as a baby or infant and subsequently found and cared for by others.

**Fig. 1:**
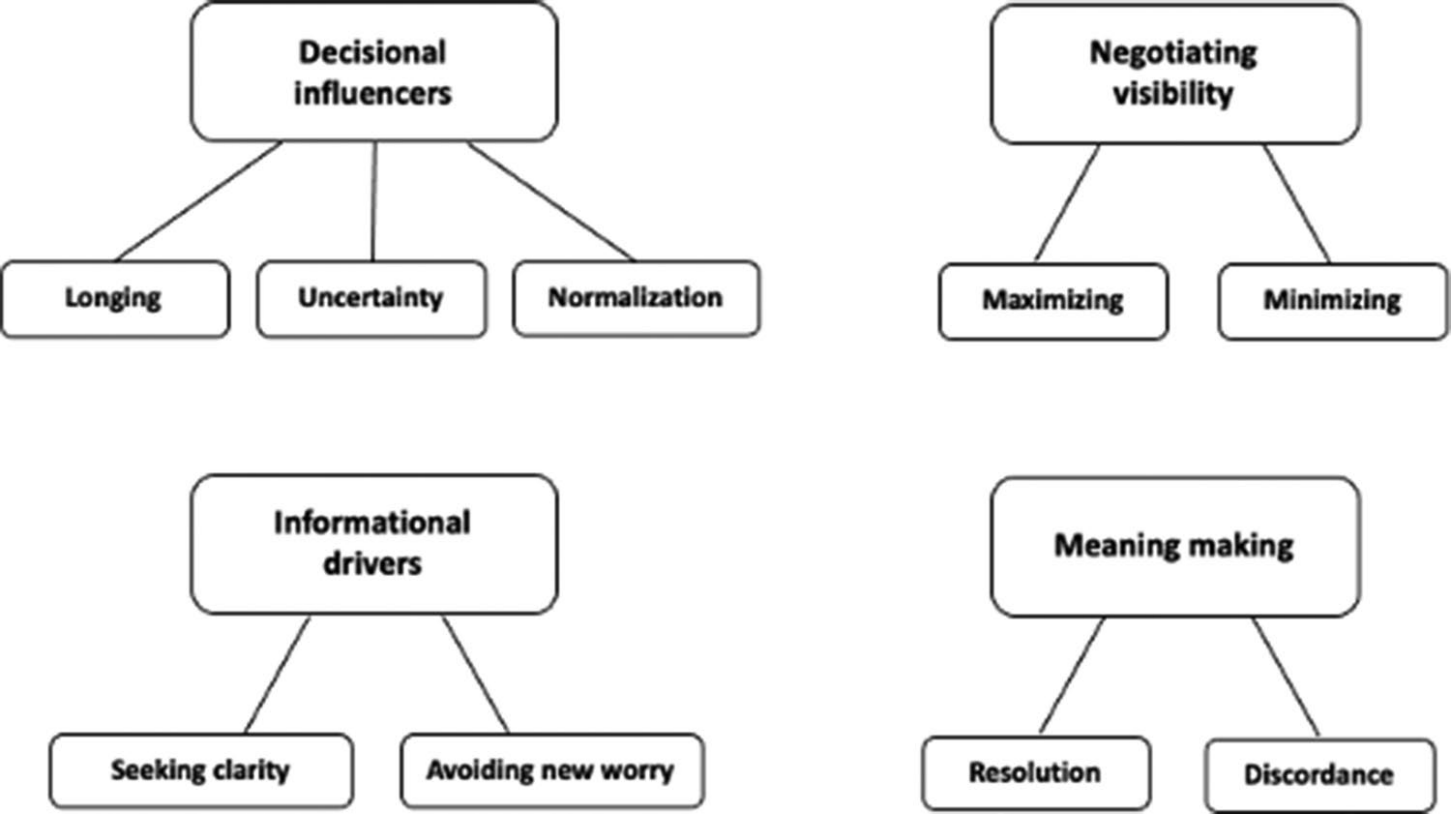
Thematic map

Analysis of the transcripts identified pre-testing themes relating to decisional influencers and informational drivers and post-testing themes of negotiating visibility and meaning making (Fig. 1).

### Decisional influencers: longing, uncertainty and normalisation

Longing, uncertainty and perceived normalisation of DTC test-taking worked as decisional influencers on UK adoptees when they were considering DTC testing. Longing pervades the transcripts, with references to a “gnawing curiosity” (Janet) and “living a bereavement” (Karen). This decisional influencer to find the missing pieces or salve a loss was not static but seemed to intensify with miscarriage, childbirth, adoptive family bereavement or other psychosocial challenges. During these periods of intensity, longing could override the otherwise careful decision-making around searching and lead to impulsive DTC test taking:

To be honest…the anxiety you feel and the desperation you feel to try and find out where you’re from can override that. I’m going to do it anyway. (Jonathan)

Working alongside these bursts in longing was the adoptees’ perception of DTC testing as offering a very quick and “all at once” route to the information they were seeking. Nonetheless, they described it as a “long shot” (Tanya), a “gamble” (Jonathan) or a “roll of the dice” (Pippa) and were very uncertain of the potential gains. Adoptees also expressed uncertainty in relation to the underlying science of the DTC testing and matching process, describing it as “technical jargon” (Tanya) and not information they could understand: “You could be speaking Swahili! I don’t get it” (Pippa). For most adoptees, this lack of understanding did not hold them back from DTC testing but neither did they resolve it, especially around what type of health information might arise from taking a DTC kit. They talked about going to their GP about other tests and procedures but were not sure they should bother a busy doctor to discuss taking a consumer DNA test that was not medically requested:

It’s not something that you can just divulge or start talking about because he’s got a room full of patients to see, so, and then I don’t know whether he is really that qualified to talk about these issues. (Tanya)

Despite their uncertainty, the perception that DTC test-taking was fairly commonplace among other adoptees, family, friends and the general public, served to offer reassurance and normalise test taking. Adoptees described seeing adverts for consumer DNA testing kits everywhere and several mentioned the gifting of DTC kits, including being prompted to use a kit because it was a gift:

I mean, it was there. It was paid for, you know. It was a present. Use it. (Stephan)

Well, it’s fascinating because that’s what my half sister and her dad, who you know, that’s what they did last Christmas…Both of them, in fairness, treated it like not very serious. So it was more of a kind of fun, a fun thing you’d do. (Sally)

Adoptees in this study described hearing or reading extensive DNA search stories in support groups and on adoptee online forums. Similarly, several participants described the visit of a DNA genealogist to an adoptee group as influencing their decision to take a DTC test. Even though the genealogists mentioned the uncomfortable information that might be found, they were seen as experts and validators of genomic consumerism, counteracting uncertainty and acting as a decisional influencer to test:

We had a big DNA speaker who talked about the experience of what you could do and what could happen and all this…in one of the meetings. And she said, if any of you are interested, she said, send me an email and I’ll send you a kit!...so I emailed her and said yes please can you send me a kit. (Dolly)

### Informational drivers: seeking clarity and avoiding worry

Information drivers is a theme relating to the type of information that adoptees in this study described seeking and why. It consists of two sub-themes: seeking clarity and avoiding new worry. Adoptees talked about seeking information from DTC testing that would help them clarify who they looked like, whether they had inherited their abilities and interests, and also information which would help them to reconcile with the circumstances of their adoption. Such clarity was seen as comforting:

It’s nice to know who you look like…if you have inherited, I don’t know, traits or whatever talents, or none. It’s nice to know, I suppose…it’s a piece of a puzzle. (Stephan)

Participants perceived DTC testing to offer empowerment in that it could potentially provide confirmatory evidence to “build a case” (Jonathan) and back-up information found via more traditional search methods.

Part of the adoptees’ mystery to solve was whether their existing health issues had genetic origins. They described how repeated experiences of being asked for family history information by healthcare providers fuelled their frustration and helplessness at not having this information. This repeated experience caused some to have to mentally prepare before going to the doctors. Adoptee status was not something that they wanted to discuss in healthcare appointments. They reported feeling uncomfortable on being quizzed as to whether they had traced their birth family or whether they were aware that they could trace:

You’d go the doctor and you’re asked if there’s any family history…I had fertility issues… and it becomes quite depressing really that you have nothing! You have nothing! People, some people, take for granted, most people take for granted. You have nothing. It’s obviously a very important question to the doctors otherwise they wouldn’t keep asking it. (Pippa)

The frustration when you go to a GP, you know: ‘Have you got this, that and the other in the family?’ And you spend the whole time saying: ‘I don’t know because I’m adopted’. You know, you’re having to disclose private stuff to people that really you shouldn’t have to, I don’t think. (Karen)

Adoptees in this study thought it would be helpful to know whether their existing health issues (e.g. unexplained infertility, eyesight issues, depression, and high blood pressure) were hereditary. Hence, the sub-theme of finding clarity represents a desire by adoptees to solve selective informational puzzles, rather than to initiate new ones.

Generally, adoptees in this study wanted to avoid information about health issues that they did not already know about. The sub-theme of “avoiding new worry” reflects the adoptees’ desire to manage impact and have some control over the type of information they were exposed to through DTC testing. There was an erroneous perception that unwanted health information could be avoided by sticking to an ancestry-focussed test rather than a health test:

I’m not interested in a health [test], I don’t want to know about things that I don’t already know about now. I’ll just wait and see what it is that gets me in the end! (Pippa)

Well because you don’t know what you might find and is it anything worse than what you’ve got? I mean, I know what I’ve got and do I really want to know anything more!? And am I brave enough to go down that road? I don’t think I am. (Tanya)

Adoptees were not unaware of the possibility of inherited predispositions towards cancer or heart disease and described the usefulness to people generally of knowing these risks, but many also said that it was personally better not to reveal these unknown risks:

I would take it too seriously…I wouldn’t be able to see it as a, you know, just a bit of fun on a, you know, I think I would probably worry about it…about how accurate it was and what I would need to do with that information, if something happened, you know, if it showed something. (Sally)

…you don’t know whether that’s come through to you or not. I mean, you could end up being a hypochondriac with all this information. You know, I don’t, if you get something wrong with you, the doctor sends you for a check-up anyway now, so. (Janet)

### Negotiating visibility: maximising and minimising

UK adoptees in this study perceived DTC testing as a means to take some control over what is visible about their origins, when it is visible and on whose terms. This was a desire for challenging the invisibility of the birth family, rather than a concern regarding the connection of their genomic and personal information per se. Gaining control over their origin information was generally accompanied by an initial maximising approach to their own visibility, in which adoptees were willing to be fully visible via DNA matching in order to gain the information they wanted. To further maximise their visibility, some participants followed their first DTC kit with further tests from rival companies or they downloaded their raw data file and uploaded it to other consumer matching databases:

I uploaded my Ancestry DNA tests wherever I could. And then I was told, ‘well, if you do 23andMe, you can upload those in other places as well. So you’ll have DNA results all over the place and you might find more birth family members, more matches. (Pippa)

Adoptees described knowingly trading the visibility of their data in order to progress their adoptee search. However, this was not necessarily a trade-off that was comfortable. Several participants were adopted foundlings and for them DTC testing offered the only means of potentially connecting with their birth family:

I did it for a reason…I had to sign up to their terms and conditions, so you know. I wanted to get what I wanted to get out of it…they want to get what they want to get out of it...I’m not going to sign up to someone’s T’s and C’s and then complain afterwards. So I went into it with my eyes open. (Robert)

I think for me, I had no choice. I, if I hadn’t been a foundling, I would never have used DNA probably, never, and I wouldn’t have said to my son, you know, have a go. (Karen)

In addition to this awareness of trade off, adoptees’ evaluations of privacy issues were positioned in the context of their existing exposure pre-DTC testing. Many had already shared their stories and some of their private details publicly online or in the press, in the hope of finding information. These experiences had primed them for the idea that maximising visibility was a necessity in birth family tracing:

I’ve done newspaper articles in Hong Kong…they’ve done a YouTube…I’ve done various things over here in the UK. So, you know, I have put myself out there a bit so I’ve got to the point now where my information is all over the place. (Tanya)

Interestingly, the desire to maximise visibility was not static. Adoptees sometimes described regretting their visibility and seeking to reduce it to manage the emotional conflict. This was about the ramifications of being seen in the online space by birth family members, who might then contact them, hide or be upset, rather than data privacy concerns regarding their genomic data being connected to their personal data. They alluded to the frequent warning adoptees receive in the broader traditional search process about treading carefully and being mindful of their impact on their birth families. They were very aware that their appearance on a DTC matching database could represent a potential “can of worms” (Stephan) for an unaware blood relative:

Well from the start people said, ‘Don’t go and knock on doors’…You don’t know what the background of that person is and what their circumstances are. Most of them are married and may have never told their spouse that they had a baby and things like that and it’s just, you know, I was just I was told you don’t do that. (Janet)

I ended up, firstly, removing my DNA from that site because I got freaked out that I’d had this match and I worried about, well, if this person figures out who I am is he going to tell my birth father? And is it going to be a grenade? So I immediately removed my profile and I hope that relative didn’t actually find out much about me. (Jonathan)

For some, the tension between longing and wanting to minimise visibility interacted with internet and social media culture to result in voyeuristic-like sleuthing. This was both perceived as a likely temptation by non-testers and also described as having been carried out by some of the test takers and also by other adoptees they knew:

They’ll do a DNA test, go onto Facebook, stalk somebody and then say, ‘I think you’re my mother or father or sister’ or whatever…So it slightly worries me that DNA is so easy to do, so accessible! And that people put so much of their lives on social media. (Pippa)

I then used that guy’s email address that I’d got when I got the email alert and I managed to find out tonnes about him. So I found out where he lived, I found out his history, I found out his Facebook, his wife…from the internet. All from Google basically…and I was able to build a family tree… (Jonathan)

Adoptees who held back from testing were concerned that visibility of DTC results to commercial third parties, particularly private healthcare or health insurance providers, could have cost implications to the adoptee.

The one and only reason I have never done it, although I’ve thought about it a few times, is they tell you that the information that you will be given will be anonymous and won’t be subject to, you know, no-one else can get this information. If you apply for it, it’s yours and yours only. Aye, not for one minute! (Carl)

I was thinking, I wonder what’s more helpful: if you say you know nothing - as in you’re adopted, I know absolutely nothing. How does that impact what price they give you? Or, what would happen…if I had the results in the bottom drawer… (Sally)

In contrast, most test-takers in this study had little to say about the implications of their genetic and personal data being held on a commercial database and what this might mean for its visibility to third parties.

### Meaning making: resolution and discordance

The theme of meaning making is about the ways adoptees in this study rationalised what they could take from their DTC test outcomes for their adoptee search, their health and their identity. This theme divides into two sub-themes of resolution and discordance. Adoptees did sometimes talk about having found the clarifying information for which they were looking. Also, the discovery that their own health issue was mirrored in a biological relative was perceived as resolving that it was not their fault after all and brought feelings of gratefulness that they themselves been able to get early help when perhaps their relative(s) had not:

The thing about all my sisters having fertility issues was quite comforting. I tend to think, well even if it’s not explained…it’s not me, if you like. It’s not something I’ve done. It’s just something that happened. (Pippa)

When I finally traced my family, my mother, I found out they had a big history of heart attacks! And I, you know, I had high blood pressure. So I feel like, oh if I wasn’t diagnosed! Maybe they had high blood pressure and weren’t diagnosed…but I felt quite good, the fact that I had been diagnosed, you know, quite early in life. (Robert)

Even new health risk information could provide a sense of closure and reassurance because their perception was that their GP could now be informed and screening could be activated:

Then I found out that my, all three of my uncles, so all three of her brothers, have had prostate cancer. And so I’ve now added that to my GP records, so that they know I now have a medical history of prostate cancer. And I’ll need to be screened when I’m early fifties…so I’ve actually found that quite empowering, rather than scary because at least I know now. (Jonathan)

Only one participant had specifically taken a consumer DNA health test, in addition to an ancestry test. Not perceiving any red flags in their results, this adoptee interpreted this as an indication of resolution of any health risk uncertainties:

I just sort of saw it as a positive and moved on, you know, with my life. You know, I’ve got no red flags here so, you know, carry on! And it’s a bit of a pat on the back. Well, not a pat on the back but it seemed like a positive, you know, to get that positive thing. Felt good! (Robert)

However, a sub-theme of discordance was also noticeable in the interview transcripts. There were ways in which the adoptees’ test outcomes did not fit with their understanding or expectations for DTC testing. Feelings of disappointment regarding the distance of the matched relatives (e.g. 4th or 5th cousins) were common and for some adoptees this fuelled further consumer DNA test taking with other companies. Feelings of frustration or sadness were expressed because not many birth relatives had taken a DNA test — something beyond the control of the participant. Adoptees described this mismatch between what they hoped for and what their results actually provided as being very challenging for themselves and other adoptees:

You always have to be mindful that whilst one, you know, you get some that are, you know, over the moon, there are many others that aren’t…go off the rails. (Tanya)

Adoptees talked about matching databases being overwhelming or the frustration of being unable to act on the information in them, perhaps because they were blocked or denied contact by matched birth family members:

The man I think is my father only died in [year] and he has a couple of brothers alive in Ireland but who am I, they’re well into their eighties. I don’t want to kill them off. So I have no right of recourse there at all. (Karen)

…my father was married, you know, with children. He was having an affair with my mum…I kind of feel guilty for doing it…even talking about it. I feel a bit sort of torn between me; I selfishly would like to meet my half-sisters.…they think their mum and dad was a certain way and then suddenly they find out that dad had two kids, you know, years ago. (Robert)

[an uncle]…sent an SMS around the, you know, side of the family saying there has to be no information handed over, no information. There’s to be no names, no dates, nothing. So all very cagey. (Stephan — informed by a matched cousin)

They also found it hard when getting their DTC results back did not resolve the issue they had hoped it would, such as fixing their depression or leading to a palatable version of their birth mother or father. This could be upsetting:

It just became a sort of, a Jerry Springer,^1^ sordid, you know, sorry tale. Um and one that I can well understand that why they invented a completely different story that was given to my adoptive parents. (Stephan)

Adoptees also described an “all-consuming” feeling that was discordant with their original perceptions of consumer DNA testing as giving adoptees more control and being less time-consuming than other search routes. They described checking their results daily and becoming aware that there was the potential for it to take over their lives. The apps and notification systems operated by the DTC testing companies were described as making it challenging to step away:

I realised the amount of time that I was starting to spend on the internet, once this thing actually came through... (Stephan)

All these Apps are just trying to make you use them more and I feel 23andMe is just like that. (Robert)

Other areas of discordance related to ethnicity estimates and unexpected information on health risks. The estimating and reporting of ethnicity by DTC testing companies, whilst sometimes providing resolution, could be discordant for some adoptees. The estimates generally appeared to cause notable confusion and in some cases frustration and identity conflict:

…where they’re getting their information from?...I mean why do they keep updating your DNA? You think when you’ve got your DNA, that’s your DNA and that’s it! I can’t understand why, you know… (Dolly)

Now you can hear I’m a London girl...protestant, London girl. My family are 100 per cent Catholic, 100 per cent Irish, genetically, and I’m struggling. I’m struggling with that a few, you know, a year on or so. I really struggle because I’m not English, I’m genetically Irish. (Karen)

Additionally, adoptees were unprepared for the health risk information that was revealed through ancestry testing and it was a cause of worry:

I think everyone has a sort of fear of cancer or an awareness around, when I found out my [birth] mum died of cancer, I thought: ‘Oh shit!’. (Robert)

I was getting heart palpitations…part of me was thinking, well I’m in my fifties, they all died in their fifties of heart problems. This is a heart thing that’s going on. (Pippa)

Sometimes they took this information to their GP, in order to clarify their risks. Only one participant was aware of genetic counselling as a profession, but all adoptees described the need for emotional and practical support in processing the meaning of the DNA results for themselves:

Then the question of course is well okay…who do you talk it through with? (Stephan)

## Discussion

This study of UK adoptees found some consistencies and also some differences to the US adoptee studies (Baptista et al. 2016; Lee et al. 2021; Strong et al. 2017), as was also the case when compared with broader recent studies on the motivations and views of the general public on DTC testing (Baig et al. 2020; Felzmann 2015; Kaufman et al. 2012; Nordgren and Juengst 2009; Saha et al. 2020; Smart el al. 2017; Strand & Källén 2021). This discussion is structured around the four themes identified in this study: (i) decisional influencers; (ii) informational drivers; (iii) negotiating visibility and (iv) meaning making in regard to the test outcomes (finding resolution or discordance).

### Decisional influencers

BAPTISTA et al.’s (2016) comparative pre-testing survey with US customers of two DTC testing companies found adoptees presented fewer positive emotions around testing than non-adoptees. The findings of this study are broadly consistent, but the qualitative approach enabled a deeper exploration of what might be influencing this difference. Adoptees described mixed feelings and a range of influences on their test decision-making, describing reticence, indecisiveness and uncertainty but combined with an intense longing to know more about their origins and biological family. This struggle is consistent with the “back and forth” identified in Strong et al.’s (2017) focus-group study (*n* = 17) of US adoptees who had yet to take a test.

A further consistent finding in relation to pre-testing views was familiarity with DTC testing. Lee et al.’s (2021) recent study found very high levels of awareness of this type of genomic consumerism among US adoptees (72.7%), with adoptees learning about DTC testing kits through social media (53%) and friends and family (36.8%). UK adoptees in this study also demonstrated high levels of awareness of test-taking among friends, family and other adoptees and referred to advertising being everywhere.

Using a semi-structured interview approach rather than a survey of closed-ended questions enabled this study to identify several novel findings. For example, DNA genealogy speakers at adoptee groups had a role in promoting and normalising consumer DNA testing as a route to information for adoptees. This was described as influencing testing by some adoptees in this sample, along with test-taking in the adoptees’ wider network and receiving a test kit as a gift. Additionally, although test-taking for the participants in this study was always part of a longer journey of reflectivity over whether to engage in the broader adoptee search, the decision to order a DTC test was often made impulsively.

Lee et al.’s (2021) recent study of adoptees, both test-takers and non-testers, found older participants were more likely to go ahead with testing. They suggest increased socioeconomic status and developmental life transitions such as birth of children as likely explanatory factors. The latter is somewhat consistent with findings in this study; significant life event such as miscarriage, childbirth, bereavement or a period of poor mental health were search triggers for participants. However, given the declining cost of testing kits in the UK,^2^ it seems more likely that the older age of test-takers is reflective of the psychosocial challenges UK adoptees face in embarking on and navigating an adoptee search rather than socio-economic cost issues.

### Informational drivers

Limited research to date has suggested that adoptees are attracted to DTC testing by the potential to obtain health information that would be useful for life goals and decision-making around, for example, reproductivity (Baptista et al. 2016; May and Grotevant 2018; Spencer et al. 2018; Strong et al. 2017). The findings of this study are not entirely consistent with this. UK adoptees did not describe seeking information for these types of decisions. The sample in this UK study is older (mean age 56.3) than for previous studies (Baptista et al. 2016: mean aged 44; Lee et al. 2021: mean age 41.6%; Strong et al. 2017: 70.6% aged < 40^3^). Therefore, participants were less likely to be using the information for such things as reproductive decision-making. However, adoptees in this study were also selective about other types of health information they were seeking, if any at all.

One participant in this study was motivated to take a specific health test, in addition to an ancestry test, although they also reported being too frightened to open the results for a week when they arrived. Other participants were less sure of the benefits to themselves of knowing new health risks. Test-takers were in the main interested to know if their existing health issues had genetic origins but wanted to avoid the worry associated with knowing about additional health risks to those they already lived with. One participant thought that so long as they avoided a health test specifically, they could avoid encountering this type of information.

The avoidance of new health risk information identified in this study is at odds with studies of US adoptees (Baptista et al. 2016; Strong et al. 2017) but is perhaps reflective of the differences in healthcare systems between the UK and USA. In this study, UK adoptees placed their trust in their general practitioner (GP) and population screening programmes, which in the UK are not restricted by fee or health insurance coverage. However, concerns that DTC test results could potentially restrict access to private medical care or insurance in the future were raised by two participants in this study who had not taken a DTC test at that time, suggesting adoptees’ views on DTC testing are influenced by their perception of the wider implications for their access to medical care. Whilst this finding is not generalisable, it is suggestive that motivations of adoptees in relation to DTC testing need to be understood within the context of the healthcare systems in the adoptee’s country of residence.

Norgren and Juengst (2009) have argued that inadequate or misunderstood genetic information might influence a person to take on a predetermined sick role in trying to adjust their self-identity. When some adoptees in this UK study did receive new family health history information, they were fearful that these new risks now predetermined their future health. It was reassurance from their NHS GP that assuaged this; otherwise, these may well have remained risks with which they continued to identify. The seeking of medical reassurance is consistent with Baptista et al. (2016) and Lee et al.’s (2021) reported findings for their US samples — 41% and 44% respectively.^4^ A novel finding from this study however is that finding a health issue in the adoptee’s birth family that was already known to the adoptee, such as issues with blood pressure or fertility, often brought comfort and alleviated self-blame. Nonetheless, there is also the potential here for misunderstanding, false reassurance, and incorrect attribution of a genetic cause.

Consistent with Baptista et al. (2016), Lee et al. (2021) and Strong et al.’s (2017) findings for US adoptees, non-health information gaps such as inherited traits and ethnicity were very important to the adoptees in this UK study. Two of the participants were foundlings^5^ from Hong Kong, subsequently adopted by families in the UK. Ethnicity results from ancestry testing were particularly important to these adoptees, including related social networking opportunities. However, ethnicity was also important to non-foundlings too, as was feeling a connection with place of origin. Some adoptees organised lone or group visits to locations near their assumed birthplace, even if the specific details were still unknown. This is consistent with Felzmann’s (2015) analysis of the “beyond familial” connections made by test-takers in the general public.

### Privacy and visibility

The findings on data privacy for UK adoptees are consistent with Baptista et al. (2016) and Strong et al. (2017) in the sense that non-test takers had concerns and test-takers raised few privacy issues. Privacy for test-takers in this study was less about data security or commercial interests and more about challenging silences or avoiding being visible to their birth relatives before they felt ready. Some adoptees described regretting their exposure and trying to hide their visibility to regain privacy. The experience of (or temptation for) voyeuristic searching was an interesting finding in this study, along with differences in the way some participants viewed their own privacy boundaries compared to those of their birth family. Whilst it is tempting to view this as driven by the adoptees’ emotional investment, this differentiated approach to privacy has also been found in the general population in a recent qualitative study (*n* = 27) of Canadian test-takers (Baig et al. 2020) and so could at least in part be reflective of broader societal trends relating to social media and internet searching. However, Baig, Mohamed and Theus’s (2020) sample was younger^6^ (aged 18–34 years compared to 39–73 years in this study) and so the relationship between age and attitudes to social media and internet searching cannot be discounted.

There was little talk of data privacy risks by adoptees in this study, a finding consistent with US studies with general population samples (Baig, Mohammed & Theus 2020: *n* = 27; Saha et al. 2020: *n* = 24), suggesting low levels of general public awareness and engagement around data privacy risks. However, adoptees did describe having already put a lot of personal information about themselves online and in the media as part of their broader adoptee search and hence already feeling like they had sacrificed their privacy. It would seem plausible, especially considering the significant public appetite for birth family reunions in the UK media, that adoptees’ prior media exposure (or observations of this among other adoptees) might be priming them to have a lower threshold for data privacy concerns when considering consumer DTC testing.

### Resolution and discordance

In this study, adoptees were keen to say that they had no regrets about having taken a DTC test, regardless of any challenges or disappointment they also described. This is consistent with the findings of Lee et al. (2021) and Baptista et al. (2016), which found 88% and 78% expressed being satisfied. However, this may not be survey information to take at face value. Baptista et al. (2016) reported some adoptees also expressed disappointment around their test results and this is consistent with the findings of this study. UK adoptees described trying to keep low expectations for their test outcomes but nonetheless, when the results arrived, they experienced feelings of disappointment and sadness, highlighting the potential psychological impact of this accessible genetic technology on this group. This finding also suggests satisfaction scores could be misleading when trying to evaluate views and experiences on genetic technologies that are not well understood by the general public.

Lee et al. (2021) found that satisfaction levels were lower regarding health information than for ancestry results. In contrast, this UK study did not find disappointment expressed in relation to health information. However, only one participant in this study had sought a specific consumer health test and for the others, new health information was not a key motivation for testing, so this result was not surprising. Rather, disappointment was often around the degree of closeness of biological relatives on the testing companies’ databases. The majority of matches that appear to customers of DTC testing will be genetic cousin relationships and these are presented as ranges (e.g. 3rd–4th cousin) (Kirkpatrick and Rashkin 2017). However, for an adoptee seeking birth parents or siblings, this genetic distance was disappointing.

Although feelings of satisfaction or disappointment for adoptees in relation to DTC testing will depend on their own individual conscious and sub-conscious hopes and needs, they will also be influenced by other factors such as whether they understand the limitations of the test. In this study, participants often referred to DTC testing as representing an objective truth, a finding consistent with other studies of public perceptions of ancestry testing (Strand & Källén 2021; Strong et al. 2017). Similarly, ethnicity results were not well understood by many participants in this study, consistent with general public reactions reported in the media (Lawton & Ifama 2018). The percentage mix of different ethnicities reported by DTC companies sometimes confused UK adoptees in this study and receiving new estimates unsettled them. Participants seemed unaware that these tests do not provide a definite ethnic identity but rather are based on a comparison between the customer’s DNA and a sample of other contemporary individuals in the company’s reference database. Each company’s reference database and algorithm changes over time, leading to changes in an individual’s ethnicity estimate (Kirkpatrick and Rashkin 2017; Strand & Källén 2021). Rather, participants in this study seemed to regard results as more definitive and struggled to reconcile updates with this understanding.

Genetic essentialism is not uncommon in studies of general public perceptions of the role of DNA in determining a person’s identity but added to this is the concern that DTC test information can be flawed, distorted or mis-interpreted (Felzmann 2015; Nordgren & Juengst 2009). It is reasonable to hypothesise that there is the potential for this to be particularly problematic for adoptees, with their lack of wider contextual birth family information. Whilst some adoptees in this study dismissed discordant or confusing ethnicity results that did not “fit” with their goals, one participant was particularly thrown by their result and really struggled to adjust their identity to include this new information. Whilst there are no studies on adoptees’ views and experiences with which to draw direct comparison, the findings that ethnicity results are either rejected as nonsensical or steadfastly accepted are consistent with Strand and Källén’s (2021) qualitative study of Swedish, British and American individuals (*n* = 14) who used DTC testing purposively to establish Viking ancestry. They concluded that for some individuals a new geneticised identity is created at the intersection between seemingly immutable DNA data and an individual’s subjective interpretation.

### Study limitations

As this is a qualitative study, these findings are not generalisable, though consistencies across the participants in this study and with the existing literature offer some support for the reliability of these findings. Although this research is concerned with the post-adoption experience, the circumstances of adoption may affect perceptions and experiences regarding DNA ancestry and health testing, and it may be that foundlings (who have no information in their official birth records) have some perceptions that are unique to them which were not identified by this study. Further research using a larger sample to understand the differences within the UK adoptee community and particularly intersectionality of characteristics is encouraged.

Additionally, self-selecting participation was unavoidable in this study because only those who felt comfortable speaking about their adoptee status were likely to agree to take part. The views of adoptees who choose not to take a DTC test have not been well explored in the existing literature and although every effort was made to obtain a more balanced sample for this study, 70% of the participants were test-takers. This compares to 80.3% test-takers in Lee et al.’s (2021) US study of adoptees and DTC testing, the only other study to date on adoptees that was designed to include both views. Additionally, double-blind coding would have been beneficial, although a sample of transcript coding was discussed with the second researcher along with the code book and revisions of the thematic map.

### Implications

Informational deficits are an obstacle to informed choice and complicated, misunderstood and potentially unreliable results could lead to inappropriate health decisions (Kaufman et al. 2012; Kirkpatrick and Rashkin 2017). Adoptees in this study did not describe having awareness of the issues around validity or reliability of health information gained via DTC tests. Pre-test genetic counselling was also unfamiliar and indeed is not common for any customers of DTC testing services (Kirkpatrick and Rashkin 2017). Yet, as this study has identified, adoptees can face strong decisional influencers such as their own longing and impulsivity and the increasing popularity of DTC testing among adoptees and in UK society generally. Therefore, information to help them make informed decisions could be beneficial. In the absence of adoptee-specific resources, disseminating AGNC resources to adoptee groups and DNA genealogists such as the leaflet: “Direct to Consumer (DTC) testing: top tips for patients” (AGNC) may be helpful.

Adoptees in this study expressed concern about receiving new health information and described worrying and seeking reassurance from their General Medical Practitioner (GP). It is important to note, however, that due to the issues with validity, sensitivity and clinical utility of DTC tests, the Royal College of General Practitioners (RCGP) has advised GPs to exercise caution when approached by their patients with their test results. They are directed to give advice based solely on family history and the usual risk assessments (RCGP & BSGM 2019). Lacking this family history, some adoptees may not therefore find reassurance via this route.

Although testing companies have encouraged a recreational understanding of their products, and indeed the participants in this study sometimes referred to DTC testing as “a bit of fun”, whether this is the case depends significantly on what the individual has at stake (Felzmann 2015). This study has highlighted that there can be complexities for adoptees in pre-test decision-making. Longing for more origin information sits alongside uncertainty around the underlying science and the adoptee’s ability to navigate the outcomes. Additionally, visibility to the birth family via matching databases opens up feelings of vulnerability. Adoptees did not express regret in undertaking DTC testing but the information they gained through the testing process was often described as disappointing or unsettling and sometimes challenging to integrate into a sense of identity. Hence, in this study, adoptees’ descriptions of their testing dilemmas or experiences do not correlate with DTC testing being singularly a fun, light-hearted experience for them.

Adoptees in this study talked about the challenge of managing their search so that it did not overwhelm them and their other life goals and commitments. However, the business model of the DTC testing companies is at odds with this challenge. Participants described needing to put things “back in the box” but struggling to do this when they received regular match notifications or hints regarding possible relations. As Felzmann’s (2015) noted in their bioethics discussion paper, encouraging ongoing continuous involvement through notifications, updates and social media is an important element of the operational strategy of some companies offering testing. It is reasonable to hypothesise that this has the potential to create an additional self-management challenge for adoptees.

In seeking to address the impact of a genetic technology on a specific group in British society (Middleton et al. 2017), the authors hope to encourage further research to inform the adoption community and support the core practice in genetic counselling of upholding individual meaning (AGNC 2019; McCarthy Veach et al. 2007). Adoptees may turn to DTC genomic testing to fill selective information gaps and create feelings of self-agency in their adoptee search. Qualitative interviews with UK adoptees indicate perceptions of gain in doing so but also the potential for unexpected information, psychosocial challenges and privacy sacrifices.

## Data Availability

The data that support the findings of this study are available from the corresponding author, [AK], upon reasonable request.

## Footnotes

1 American reality talk show featuring mature topics and confrontation between guests.

2 At the time of writing, an Ancestry.com DNA testing kit could be purchased in the UK at £59 + shipping. Available at https://www.ancestry.co.uk/

3 Mean age is not given by the authors and reported age is displayed in ranges.

4 Forty-one percent of all participants in Baptista et al. (2016) and 44% of those participants who received health risk information (Lee et al. 2021).

5 Foundlings, having been abandoned, have no information on either birth parent.

6 Mean age is not reported and calculation was not possible as individual ages were not reported (Baig et al. 2020).
